# Association between surgically treated knee injury and knee arthroplasty: an explorative study based on Finnish nationwide register-based data

**DOI:** 10.2340/17453674.2026.46170

**Published:** 2026-07-02

**Authors:** Olli HEIKKILÄ, Reijo SUND, Heikki KRÖGER, Antti JAROMA, Joonas SIROLA

**Affiliations:** 1 Kuopio University Hospital, Kuopio; 2 University of Eastern Finland (UEF), Kuopio Musculoskeletal Research Unit (KMRU), Kuopio, Finland

## Abstract

**Background and purpose:**

Knee injuries are established risk factors for knee osteoarthritis (OA). We aimed to investigate whether surgically treated knee injuries are associated with an increased incidence of end‑stage knee OA necessitating knee arthroplasty (KA).

**Methods:**

Using nationwide Finnish registers, we identified all individuals aged 15–99 years who underwent surgery for knee injuries between 1986 and 2024. Injuries were categorized as knee fracture surgeries or non‑fracture knee injury surgeries. The date of the first identified injury surgery served as the index point from which person years under the exposure of interest were accrued. The control population comprised all Finnish residents without a recorded knee injury diagnosis and surgical treatment. Diagnoses and procedures were retrieved from the Care Register for Health Care (CRHC); KAs were identified from the Finnish Arthroplasty Register (FAR) and CRHC. Incidence rates of KA were calculated for both injury categories and the control population, stratified by age, sex, and year. Incidence comparisons between exposure and control groups were performed using age‑, sex‑, and year-standardized standardized incidence ratios (SIRs).

**Results:**

In the fracture surgery cohort, there were 2,163 KAs with over 489,000 person‑years of follow‑up. In the non‑fracture injury surgery cohort there were 13,570 KAs with over 3.01 million person‑years of follow‑up. In the control population there were 205,440 KAs with over 167 million person‑years of follow‑up. Relative to the corresponding general population, the incidence of KA was increased in both injury categories, non-fracture injury surgery SIR 3.74 (95% confidence interval [CI] 3.68–3.80) and fracture surgery SIR 2.08 (CI 1.99–2.17). The greatest excess was among the youngest group (15–54 years): fracture surgery SIR 8.12 (CI 7.40–8.89); non‑fracture injury surgery SIR 6.05 (CI 5.84–6.27).

**Conclusion:**

Surgically treated knee injuries were associated with an increased incidence of KA, most notably in those aged 15–54 years. In older age groups, the additional effect of the injury surgery appears smaller, likely because age itself markedly increases the baseline incidence of KA.

Established risk factors for knee osteoarthritis (OA) include female sex, increasing age, overweight, and prior knee injury [1-4]. Traumatic knee injuries increase the risk of subsequent knee OA [5]. Prior studies have reported approximately 4‑fold odds after anterior cruciate ligament (ACL) injury and 6‑fold odds after meniscal injury [6]. Proximal tibial and patellar fractures have also been strongly associated with later knee OA [7]. Management of knee OA is primarily nonoperative, including non-pharmacological treatments like education, exercise, and lifestyle changes combined with analgesia if needed [8]; when symptoms persist despite nonoperative care and there is defined radiographic knee OA, knee arthroplasty (KA) is the treatment of choice [9].

A limited number of studies have assessed the risk of KA following specific surgically treated injuries such as ACL reconstruction or proximal tibial fracture reconstruction (PTFr) [10-12]. Previous studies have reported an increased risk of KA after ACL reconstruction, with relative risks (RR) ranging from 3.3 to 20 fold depending on age [10,11]. A 3.2 fold increased risk of total knee arthroplasty (TKA) has been observed after PTFr [12]. Additionally, arthroscopic partial meniscectomy has been associated with a 10 fold RR of KA [13].

Evidence has largely examined single injury types in isolation. Given the strong association between a variety of knee injuries and knee OA [14], a population‑based assessment of KA incidence following different surgically treated knee injuries is warranted to refine the epidemiological picture.

We aimed to examine whether surgically treated knee injuries are associated with increased incidence of KA in specific age and sex groups, compared with the corresponding knee‑injury-surgery‑free Finnish population.

## Methods

### Study design and patients

This study was a retrospective descriptive Finnish nationwide register-based study, comparing the incidence of KA among individuals with surgically treated knee injuries and the knee-injury-surgery–free general population, using standardized incidence ratios (SIRs).

The study is reported according to STROBE guidelines.

This register‑based study included knee injuries, surgical procedures, and KAs (unicompartmental or total, excluding high tibial osteotomies) performed in Finland between 1986 and 2024. Previous knee injuries and surgical treatments were identified using ICD‑8/9/10 diagnostic codes and FIHW procedure codes (1986–1996 Hospital League; 1996–2024 Nomesco’s Finnish version). Inclusion in the injury groups required both a defined knee injury diagnosis and a corresponding surgical procedure (see Supplementary data). Conservatively treated injuries were excluded from injury groups, as surgically treated injuries are generally more severe and more often result from high‑energy trauma. Combining diagnostic codes with procedural codes thus improves the accuracy of exposure classification compared with diagnostic codes alone.

We divided the whole Finnish population into different risk groups: (i) people with knee fracture surgery without pre-existing knee OA, (ii) people with knee non-fracture injury surgery without pre-existing knee OA, (iii) people with knee fracture surgery who have knee OA, (iv) people with knee non-fracture injury surgery who have OA, and (v) the rest of the population which comprised people without surgically treated knee injuries. The fracture surgery group comprised proximal tibial and fibular, distal femoral, and patellar fractures; the non-fracture injury surgery group comprised ligamentous, chondral, meniscal, and muscle/tendon injuries, as well as patellar dislocations (see Supplementary data). Each participant was assigned to a single group based on only the first recorded knee injury and surgery; in cases with multiple procedures, classification was based on the primary treatment code. Individuals with pre‑existing knee OA (groups iii and iv) were excluded from the injury groups to better assess the potential causal relationship between surgically treated injury and subsequent end‑stage OA. KA events and person years of follow up were then calculated for each group and stratified by sex, age, and calendar year.

Population counts were obtained from Statistics Finland’s StatFin database [17]. Person-years accrued from the study start date, the date of birth, or the date of the first identified injury and surgery, whichever occurred last, until KA, death, or December 31, 2024, whichever occurred first. The study focused on adults; thus, the youngest age category comprised post‑pubertal individuals (15–54, 55–64, 65–74, and 75–99 years).

### Data sources

Data was obtained from the PERFormance, Effectiveness and Cost of Treatment episode (PERFECT) project, maintained by the Finnish Institute for Health and Welfare (FIHW). The project monitors specialized healthcare treatments that are costly and affect large patient groups. For this study, we used data from the Care Register for Health Care (CRHC) and the Finnish Arthroplasty Register (FAR). The CRHC systematically records surgical procedures and treatments, including KAs, and the FAR has maintained near‑complete coverage for primary KAs throughout its operation [16-18]. Together, these nationwide registers provide high coverage and completeness of the data, minimizing the risk of missing cases.

### Statistics

Analyses were performed using R (version 4.3.3; package popEpi; R Foundation for Statistical Computing, Vienna, Austria). KAs were identified from FAR and CRHC for both injury and control groups. KAs in the control group were derived by subtracting knee-injury‑surgery-related KAs from the total number of KAs. Incidences (per 100,000 person‑years) were estimated by relating the number of KAs to follow‑up time for each group. For each year, control group person‑years were based on the mid‑year population of Finland; person‑years contributed by injury cohorts were excluded from the corresponding general population denominators. Age‑, sex‑, year-, and group-standardized SIRs were used to assess whether KA incidence was elevated in injury cohorts compared with the general population. Expected KAs in injury cohorts were obtained by multiplying the control population’s KA incidence by the total follow‑up time accrued by the injury cohorts. SIRs were calculated by dividing observed by expected KAs within each exposure, age, and sex stratum. 95% confidence intervals (CIs) were calculated for SIRs. An SIR > 1 signifies that the observed incidence in the exposure group is higher than expected based on the general population; an SIR < 1 indicates a lower-than expected incidence; and an SIR = 1 denotes equivalence.

### Ethics, funding, data sharing plan, use of AI tools, and disclosures

The Finnish Institute for Health and Welfare granted permission for the use of PERFECT data (THL/7019/6.02.00/2020). No external funding was received. The original data cannot be shared due to privacy regulations. Copilot was used solely for language editing. The authors declare no conflicts of interest. Complete disclosure of interest forms according to ICMJE are available on the article page, doi: 10.2340/17453674.2026.46170

## Results

Between 1986 and 2024, in the fracture surgery cohort there were 2,163 KAs (776 in men and 1,387 in women) over 489,000 person‑years of follow‑up. In the non‑fracture injury surgery cohort there were 13,570 KAs (7,035 in men and 6,535 in women) with over 3.01 million person‑years of follow‑up. In the control population there were 205,440 KAs (67,691 in men and 137,749 in women) with over 167 million person‑years of follow‑up (Figure). Counts of KAs and person‑years by age and sex are presented in Table 1.

**Figure:**
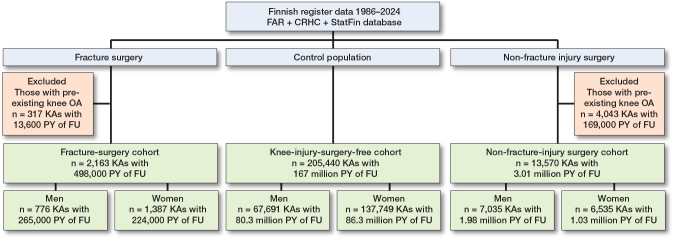
Flow chart of the study. FAR = the Finnish Arthroplasty Register; CRHC = the Care Register for Health Care; StatFin = Statistics Finland; KA = knee arthroplasty; OA = oateoarthritis; PY = person-years; FU = follow-up.

**Table 1:** Number of knee arthroplasties (KAs) and follow-up times in person-years for both sexes, for every age group and with non-stratification in the knee fracture surgery cohort, non-fracture injury surgery cohort and general population in Finland during the study period of 1986 to 2024

	KAs after fracture surgery ^a^	KAs after non-fracture-injury surgery ^b^	KAs in general population	Person-years (x 10^3^) in non-fracture-		
				fracture surgery ^a^	injury surgery ^b^	general population
Men	776	7,035	67,691	265	1,977	80,270
Women	1,387	6,535	137,749	224	1,028	86,257
Total	2,163	13,570	205,440	489	3,005	166,527
Age groups						
15–54	460	3,023	15,608	230	2,081	106,985
55–64	573	5,387	50,615	92	547.3	24,518
65–74	678	3,773	80,279	84	277.7	19,499
75–99	452	1,387	58,938	82	98.8	15,525
Age groups – men						
15–54	263	1,712	5617	162	1,424	54,147
55–64	242	2,969	18,641	50	343.5	11,895
65–74	193	1,803	26,740	34	160.7	8,738
75–99	78	551	16,693	18	49.7	5,490
Age groups – women						
15–54	197	1,311	9,991	68	657.8	52,838
55–64	331	2,418	31,974	42	203.8	12,622
65–74	485	1,970	53,539	50	117.0	10,761
75–99	374	836	42,245	64	49.1	10,035

a Surgically treated proximal tibial and fibular, distal femoral, and patellar fractures.

b Surgically treated knee ligament, cartilage, meniscus, muscle/tendon injuries, and patellar dislocations.

### Incidence of KA

Overall, non‑fracture injury surgeries were associated with a higher incidence of KA (SIR 3.74, CI 3.68–3.80) than fracture surgeries (SIR 2.08, CI 1.99–2.17) relative to the general population (Table 2).

**Table 2:** Assessment of the incidence of knee arthroplasty (KA) following surgically treated knee injuries in Finland during 1986 to 2024, by using the standardized incidence ratio (SIR) method. A SIR greater than 1 indicates an increased incidence of KA compared with the general Finnish population of the same sex

Sex	Fracture surgery ^a^ SIR (CI)	Non-fracture-injury surgery ^b^ SIR (CI)
Men	2.44 (2.28–2.62)	3.90 (3.81–4.00)
Women	1.92 (1.82–2.02)	3.57 (3.49–3.66)
Both	2.08 (1.99–2.17)	3.74 (3.68–3.80)

CI: 95% confidence interval

a and b See Table 1.

The incidence varied by age and was highest in those aged 15–54 years: fracture surgery SIR 8.12 (CI 7.40–8.89) and non‑fracture injury surgery SIR 6.05 (CI 5.84–6.27). SIRs decreased progressively with increasing age across both injury categories and both sexes. Non‑fracture injury surgery was associated with a higher SIR than fracture surgery in all age categories except the youngest (Table 3).

**Table 3:** Assessment of the incidence of knee arthroplasty (KA) following surgically treated knee injuries in Finland during 1986 to 2024, by using the standardized incidence ratio (SIR) method. A SIR greater than 1 indicates an increased incidence of KA compared with the general Finnish population

Age group	Fracture surgery ^a^ SIR (CI)	Non-fracture-injury surgery ^b^ SIR (CI)
15–54	8.12 (7.40–8.89)	6.05 (5.84–6.27)
55–64	2.48 (2.28–2.69)	3.86 (3.76–3.97)
65–74	1.69 (1.57–1.82)	3.02 (2.92–3.11)
75–99	1.29 (1.17–1.41)	2.86 (2.71–3.01)

CI: 95% confidence interval

a and b See Table 1.

Among men aged 15–54 years in the fracture surgery cohort, the SIR was 9.12 (CI 8.07–10.3), the highest observed in any subgroup. The corresponding SIR for women of the same age was lower, though still the highest within female age strata (SIR 7.08, CI 6.14–8.12). In all other age categories, women exhibited higher SIRs than men (Table 4).

**Table 4:** Assessment of the incidence of knee arthroplasty (KA) following surgically treated knee injuries in Finland during 1986 to 2024, by using the standardized incidence ratio (SIR) method. A SIR greater than 1 indicates an increased incidence of KA compared with the general Finnish population of the same sex

Sex Age group	Fracture surgery ^a^ SIR (CI)	Non-fracture-injury surgery ^b^ SIR (CI)
Men		
15–54	9.12 (8.07–10.3)	6.38 (6.09–6.69)
55–64	2.40 (2.11–2.72)	4.01 (3.87–4.16)
65–74	1.56 (1.35–1.80)	3.03 (2.89–3.17)
75–99	1.21 (0.96–1.45)	2.79 (2.57–3.03)
Women		
15–54	7.08 (6.14–8.12)	5.67 (5.37–5.98)
55–64	2.54 (2.27–2.82)	3.70 (3.55–3.85)
65–74	1.75 (1.60–1.91)	3.01 (2.87–3.14)
75–99	1.30 (1.18–1.44)	2.90 (2.71–3.11)

CI: 95% confidence interval

a and b See Table 1.

For non‑fracture injury surgery, men had higher SIRs than women in all age categories except the oldest (see Table 4).

## Discussion

We examined whether surgically treated knee injuries confer an increased incidence of KA relative to the Finnish population without recorded surgically treated knee injuries. We observed a 4‑fold increase after non‑fracture injury surgery and a 2‑fold increase after fracture surgery, with the greatest excess among those aged 15–54 years (6‑fold and 8‑fold, respectively).

The pronounced incidence in the youngest group likely reflects several mechanisms. First, primary knee OA leading to KA is comparatively uncommon at younger ages; therefore, a prior injury constitutes a more prominent contributor to incidence. Second, intra‑articular fractures may damage cartilage and increase joint loading, predisposing to OA [19]. Third, ligamentous injuries may leave residual instability despite surgical treatment [20]. A combination of post‑traumatic cartilage damage, mechanical instability, and high activity levels may underlie the elevated incidence in younger adults.

SIRs declined with increasing age, consistent with the rising baseline incidence of KA with age; the proportional contribution of prior injury therefore appears smaller in older strata. Prior studies of ACL reconstruction also reported greater relative risks in younger cohorts [10,11]. In contrast, studies of proximal tibial fractures have suggested that higher age is associated with increased absolute risk of KA [12,21], which does not contradict our findings given the different denominators and effect measures (population‑based SIRs vs individual‑level hazard ratios).

Our finding of a 6‑fold SIR in 15–54‑year‑olds with prior non‑fracture-injury surgery aligns with Visnes et al., who reported a roughly 3‑fold RR of KA in 30–39‑year‑olds after ACL reconstruction [10], and with Abram et al., who observed even higher RRs in younger adults [11]. However, their approaches differed from ours: Abram et al. calculated RRs using contemporary counts of KA among patients with prior ACL reconstruction vs the general population in 2016–2017, whereas our SIRs are based on accumulated person‑years from 1986 to 2024, providing robust incidence estimates for both exposure and control populations.

In our data, men had higher SIRs than women after non‑fracture injury surgery, except in the oldest category, whereas prior studies have reported higher hazards for women after ACL reconstruction or arthroscopic partial meniscectomy [10,11,13]. Because those studies assessed cumulative risks or hazards for selected surgical cohorts and we estimated population‑based SIRs, direct comparisons are not straightforward. Conversely, after fracture surgery, women had higher SIRs than men in every category except the youngest; this aligns with reports that women are more likely to undergo KA after proximal tibial fractures [12,21]. Additional investigation is required to determine the underlying mechanisms responsible for the disparity in SIRs between sexes. Our overall SIR for fracture surgery is lower than prior hazard‑ratio estimates [12], likely reflecting methodological and cohort differences, and the inclusion of a broader set of fracture types in our study.

In the youngest age stratum (15–54 years), the SIR for KA was higher following fracture surgery than after non fracture-injury surgery. In contrast, when considering all ages combined, non fracture-injury surgeries were associated with a higher overall SIR than fracture surgeries. A plausible explanation is that younger individuals more frequently sustain high energy fractures that typically require operative treatment and often involve intra articular damage, accelerating the development of knee OA [22]. In older age groups, fractures are more often low energy injuries that may be managed conservatively, resulting in a lower long term risk of arthrosis and KA and to be determined to control group in our study [22]. Conversely, surgeries for non fracture injuries may leave residual mechanical instability or altered joint loading, which contributes to a more gradual but consistently elevated risk of knee OA across all age strata [20].

### Strengths

Key strengths include the large Finnish nationwide register‑based cohort and long observation period. The CRHC systematically records surgical procedures and treatments, including KAs, and the FAR has maintained near‑complete coverage for primary KAs throughout its operation [18].

The study population represents an unselected, nationwide Finnish population spanning a long observation period. All individuals received care according to standardized Finnish clinical and procedural practices, independent of patient background or demographics. Consequently, the overall generalizability of the results is high. The external validity of the findings in other countries is comparable to that typically associated with Finnish healthcare registry data.

### Limitations

Limitations include potential residual confounding by conditions associated with OA risk (e.g. inflammatory arthritides) that were not excluded [23]. However, such conditions are present in both injury and control populations, which likely attenuates, rather than inflates, between‑group differences.

Variations in diagnostic and surgical practices for knee injuries over the study period may have introduced bias, as fluctuations in the identification of surgically treated knee injuries could lead to either over or under ascertainment of cases. However, temporal changes in the diagnostic criteria for knee OA and in KA procedures are unlikely to influence the main findings, because any potential loss or variation in diagnostic data would affect both the injury and control groups in a comparable manner, thereby preserving the relative incidence estimates between the groups. Some controls may have sustained unrecorded or undiagnosed knee injuries for which surgical treatment was possibly needed, potentially biasing associations towards the null.

Our study was an explorative register study comparing the incidence of KA in specific age and sex groups after surgically treated knee injury related to Finnish citizens without such surgeries. Thus, those who had a knee injury without surgical treatment are part of the control group.

### Conclusion

Surgically treated knee injuries were associated with an increased incidence of KA across all age groups and in both sexes compared with the corresponding general population. The incidence was highest among those aged 15–54 years: approximately 8‑fold after fracture surgery and 6‑fold after non‑fracture injury surgery.

### Supplementary data

Supplementary Table 1 is available as Supplementary data on the article homepage, doi: 10.2340/17453674.2026.46170

## Supplementary Material



## Notes

HO, SR, KH, JA, SJ: writing the manuscript. HO, SR, KH, JA, SJ: data analysis and interpretation of the results. SR, KH, JA, SJ: supervision of the study and proofreading.

Handling co-editors: Li Felländer-Tsai and Robin Christensen

*Acta* thanks Lukas Berglund and Lina Holm Ingelsrud for help with peer review of this manuscript.
