# Supplementary material for: Association between surgically treated knee injury and knee arthroplasty: an explorative study based on Finnish nationwide register-based data
Source: Acta Orthop. 2026 Jul 2;97:465–70. doi: 10.2340/17453674.2026.46170 (PMC13324889; doi:10.2340/17453674.2026.46170)
Supplement: Supplementary file 1 [file ActaO-97-46170-s1.pdf]

List of diagnosis and treatment codes used in the study

Group (1) included proximal tibial and fibular, distal femoral and patellar fractures
Diagnoses:

ICD-10

S72.4 Fracture of the distal femur
S82.0 Fracture of the patella
S82.1 Fracture of the proximal tibia
S82.7 Multiple fractures of the knee and/or lower leg
S82.8 Fracture of other parts of the knee or lower leg
S82.9 Unspecified fracture of the knee and/or lower leg
S87 Crushing injury of the knee and/or lower leg

ICD-9

8212A Fracture of the lateral or medial femoral condyle, uncomplicated
8212B Fracture of the distal epiphysis, uncomplicated
8212X Fracture of other parts of the distal femur, uncomplicated
8213A Fracture of the medial or lateral femoral condyle, complicated
8213B Fracture of the distal epiphysis, complicated
8213X Fracture of other parts of the distal femur, complicated
8220A Fracture of the patella, uncomplicated
8221A Fracture of the patella, complicated
8230A Fracture of the proximal tibia or fibula, uncomplicated
8231A Fracture of the proximal tibia or fibula, complicated

ICD-8

821.21 Fracture of the distal femur, uncomplicated
821.31 Fracture of the distal femur, complicated
822.0 Fracture of the patella, uncomplicated
822.10 Fracture of the patella, complicated

Group (2) included knee ligament, cartilage, meniscus, muscle/tendon injuries and patella dislocations

Diagnoses:

ICD-10

S76.1 Injury of the quadriceps muscle or tendon
S83 Dislocation, sprain, and/or strain of joints and ligaments of the knee
S86.7 Injury of multiple muscles or tendons in the knee or lower leg region

S86.8 Injury of other muscle or tendon in the knee or lower leg region
S86.9 Injury of unspecified muscle or tendon in the knee or lower leg region

ICD-9

8360A Rupture of the medial meniscus
8361A Rupture of the lateral meniscus
8362A Rupture of other meniscus or cartilage
8363A Dislocation of the patella, uncomplicated
8364A Dislocation of the patella, complicated
8365A Dislocation of the knee, uncomplicated
8365B Dislocation of the knee, uncomplicated, with nerve or vascular injury
8366A Dislocation of the knee, complicated
8366B Dislocation of the knee, complicated, with nerve or vascular injury
8438A Injury of thigh muscles
8440A Injury of the lateral collateral ligament of the knee
8441A Injury of the medial collateral ligament of the knee
8442A Injury of the anterior cruciate ligament of the knee
8442B Injury of the posterior cruciate ligament of the knee
8443A Injury of the superior tibiofemoral joint
8448A Multiple ligament injury of the knee
8449X Knee injury, not otherwise specified

ICD-8

836.00 Dislocation of the patella, uncomplicated
836.10 Dislocation of the patella, complicated
844.00 Distortion and strain of the knee and lower leg
836.01 Dislocation of the knee, unspecified, uncomplicated
836.11 Dislocation of the knee, unspecified, complicated

Between years 1996 and 2024 Nomesco's treatment codes:

NFJ Femoral fracture surgeries
NFK Femoral bone surgeries
NFL Thigh muscle or tendon surgeries
NGD Knee meniscus surgeries
NGE Knee ligament and capsule surgeries
NGF Knee synovial membrane / cartilage surgeries
NGG Knee reconstructive surgeries and arthrodesis
NGH Knee joint / patellar reduction and other procedures
NGJ Lower leg / patellar fracture surgeries
NGK Lower leg and patellar bone surgeries
NGL Lower leg muscle and tendon surgeries

Between years 1986 and 1996 Hospital League treatment codes:

1221 Arthroscopy and meniscus surgery
1223 Arthroscopy and cartilage surgery
1224 Arthroscopy and removal of loose body
1225 Arthroscopy and other therapeutic procedure
9123 Closed reduction and casting in the operating room or under anesthesia
9124 Change of cast, wedging, or equivalent procedure in the operating room or under anesthesia
9126 Closed reduction and osteosynthesis
9127 Open reduction, or reduction without osteosynthesis, with casting or equivalent
9128 Open reduction and osteosynthesis
9131 Reconstruction of the joint surface (includes bone grafting)
9139 Other procedure related to this group
9163 Delayed osteosynthesis
9165 Removal of osteosynthesis material
9231 Closed reduction of a dislocated joint
9232 Surgical repair of knee ligament and/or capsule injury
9243 Plastic reconstruction of the patella or its capsule and tendons
9244 Ligament plastic surgery or reconstruction of the knee not involving the patella
9251 Joint aspiration or lavage under anesthesia
9254 Arthrotomy
9255 Meniscectomy
9257 Operative mobilization and possible soft tissue plastic surgery
9281 Knee arthrodesis
9292 Femoral osteotomy
9311 Knee debridement
9315 Patellectomy
9371 Procedures involving the knee extensors
9372 Procedures involving the knee flexors
9373 Procedures involving the knee joint
9411 Osteotomies of the knee region
9412 Repair of the knee extensor mechanism
9413 Surgical repair of knee instability
